# Pharmacokinetic Interaction between Metformin and Verapamil in Rats: Inhibition of the OCT2-Mediated Renal Excretion of Metformin by Verapamil

**DOI:** 10.3390/pharmaceutics12050468

**Published:** 2020-05-21

**Authors:** Seung Yon Han, Young Hee Choi

**Affiliations:** College of Pharmacy and Integrated Research Institute for Drug Development, Dongguk University_Seoul, 32 Dongguk-lo, Ilsandong-gu, Goyang-si 10326, Korea; hsyglory@gmail.com

**Keywords:** metformin, verapamil, drug interaction, organic cation transporter 2, renal excretion

## Abstract

The incidence of hypertension in diabetic patients has been increasing and contributing to the high mortality of diabetic patients. Recently, verapamil use was found to lower fasting blood glucose levels in diabetic patients, which led to a new indication of verapamil as combination treatment with anti-diabetic agents such as metformin. As pharmacokinetic (PK) interaction can affect drug efficacy and safety in drug combination, their PK-based interaction is recommended to be evaluated in preclinical levels as well as clinical levels. In case of metformin and verapamil, organic cation transporter (OCT) 1 and 2 primarily mediate metformin distribution to the liver and its elimination into urine, whereas cytochrome P450 is responsible for the hepatic metabolism of verapamil. Verapamil is also known as a potential OCT2 inhibitor. Thus, PK interaction between metformin (30 mg/kg) and verapamil (20 mg/kg) were investigated after their simultaneous administration to rats. In our results, verapamil inhibited the OCT2-mediated renal excretion of metformin, subsequently leading to increase of the systemic exposure of metformin. In contrast, metformin did not influence the pharmacokinetic pattern of verapamil. Although the further clinical investigation is required, our finding suggests a possibility of OCT2-mediated interaction of metformin and verapamil.

## 1. Introduction

The incidences of diabetic mellitus and hypertension are rapidly growing worldwide, and hypertension is an important cardiovascular risk factor in patients with type 2 diabetes mellitus (T2DM) [1,2,3]. The dramatically increased (two- to four-fold) risk of cardiovascular diseases in T2DM patients is their leading cause of mortality [4,5,6]. Insulin resistance and compensatory hyperinsulinemia are also frequent findings in hypertensive patients [7,8]. Since blood pressure-lowering treatment is important to reduce the risk of developing cardiovascular complications in T2DM patients, a combination of anti-diabetic and anti-hypertension drugs is needed and increasingly used [9].

Metformin, an oral biguanide antihyperglycemic agent, increases hepatic and skeletal muscle insulin sensitivity and decreases hepatic glucose production without causing hypoglycemia [10,11,12]. Metformin also reduces cardiovascular disease complications such as high blood pressure in patients with type 2 diabetic mellitus (T2DM) [3,13,14]. Metformin pharmacokinetics (PK) and pharmacodynamics (PD) rely on the organic cation transporter (OCT) 1 in the sinusoidal membrane of hepatocytes to transport metformin into the liver, its pharmacological target site. The metformin concentration in the liver determines the metformin’s effect on inhibiting glucose production [15,16,17]. OCT2 in the basolateral membrane of the renal proximal tubules mediates the renal excretion of metformin as ~70% of the metformin dose [16,18]. Reduced renal excretion of metformin via renal OCT2 inhibition causes an increase of systemic exposure (i.e., plasma concentration of metformin), which can result in lactic acidosis [19]. Previous reports that metformin suppresses P-glycoprotein (P-gp) and pregnane X receptor (PXR)-regulated transactivation of the cytochrome P450 (CYP) 3A4 gene [20,21] suggest that the potential for a PK-based drug-drug interaction (DDI) occurrence between metformin and verapamil.

Verapamil, as a calcium channel blocker, is recently emerged as a combination drug in DM patients with hypertension, because verapamil lowers fasting blood glucose levels and enhances insulin secretion in T2DM patients [22,23]. In particular, it is an important issue to regulate insulin secretion as a compensatory mechanism of hyperinsulinemia in hypertensive patients [7,8]. Verapamil has been popularly used for treatment of hypertension and supraventricular tachyarrhythmias [22,23,24,25,26,27]. Orally administered verapamil is rapidly and well-absorbed, and then it is eliminated via extensive cytochrome P450 (CYP)-mediated hepatic metabolism [25,26,27,28,29]. An *N*-methylated metabolite, norverapamil, has been shown to have a vasodilator effect in vitro [13]. Verapamil also has an inhibitory effect on P-gp and OCT2 in in vitro and clinical studies [16,30,31]. Although it was reported that verapamil reduced the maximum blood glucose concentration and area under the blood glucose concentration-time curve in healthy adults orally administered with metformin and verapamil together [32], there was no direct measurement of metformin concentration in liver, which may be a substantial evidence on its glucose tolerance activity. Moreover, there has been no report to explain whether transporter and/or metabolic enzyme-mediated PK changes of metformin and verapamil in their combination. In the meantime, new findings regarding positive effect of verapamil for the treatment of hypertension in DM patients have been introduced [22,23], there is a need to investigate how the plasma and tissue concentrations of metformin and verapamil change in DDI events, especially at preclinical levels [33,34]. Since changes in plasma and tissue concentrations of these drugs in combination are associated with their efficacy and toxicity [35,36], the PK interactions of metformin and verapamil were evaluated in rats based on their plasma and tissue concentration changes.

## 2. Materials and Methods

### 2.1. Chemicals

Metformin hydrochloride was donated from Daelim Pharmaceutical Company (Seoul, Korea). Verapamil hydrochloride, norverapamil, ipriflavone (internal standard (IS) for the high-performance liquid chromatography (HPLC) analysis of metformin) and propranolol (IS for the HPLC analysis of verapamil and norverapamil), the reduced form of β-nicotinamide adenine dinucleotide phosphate, tris(hydroxymethyl)-aminomethane (Tris) buffer and ethylenediamine tetraacetic acid were purchased from Sigma–Aldrich Corporation (St. Louis, MO, USA). HEK-293 cells overexpressing OCT1 (SLC22A1) and OCT2 (SLC22A2) were purchased from Corning Life Sciences (Corning, NY, USA). Other chemicals were of reagent or HPLC grade.

### 2.2. Animals

The protocol for the animal studieswas approved by the Animal Care and Use Committee of the College of Pharmacy, Dongguk University-Seoul, Korea (approval no. IACUC-2013-006, 15 December 2013). Male Sprague–Dawley rats (5–7 weeks old, weighing 190–260 g) were purchased from Taconic Farms Inc. (Samtako Bio Korea, O-San, Korea). The rats were housed and handled similarly based on the reported methods [17,33,34].

### 2.3. Pharmacokinetic Studies of Metformin, Verapamil and Both Drugs in Rats

Early in the morning, the carotid artery (for blood sampling in the intravenous and oral studies) and the jugular vein (for intravenous drug administration only in the intravenous study) of the rats were cannulated, similar to previously reported methods [17,33,34]. The rats were not restrained during the experimental period.

For the intravenous study, 30 mg (2 mL)/kg of metformin (as metformin hydrochloride dissolved in 0.9% NaCl-injectable solution; *n* = 6), 20 mg (2 mL)/kg of verapamil (as verapamil hydrochloride dissolved in 0.9% NaCl-injectable solution; *n* = 6) and both drugs together (*n* = 6) were administered to the rats. Blood samples (approximately 0.12 or 0.22 mL for each drug alone or both drugs together, respectively) were collected via the carotid artery at 0, 1, 5, 15, 30, 60, 90, 120, 180, 240, 300 or 360 min after the start of the drug administration. After each blood sampling, 0.3 mL of 0.9% NaCl-injectable solution containing heparin (20 U/mL) was immediately flushed into each cannula to prevent blood clotting. The blood samples were immediately centrifuged and a 50 µL (or two 50 µL for both drugs) of plasma sample was stored at −80 °C (Revco ULT 1490 D-N-S; Western Mednics, Asheville, NC, USA). The 24-h urine sample (Ae_0–24 h_) and the gastrointestinal tract (including its contents and feces) sample at 24 h (GI_24 h_) were prepared following previously reported methods [17,33,34], and the samples were also stored at −80 °C.

For the oral study, after overnight fasting with free access to water, the same doses of metformin (*n* = 5), verapamil (*n* = 5) and both drugs together (*n* = 5) were orally administered (total oral volume of 6 mL/kg) to the rats using a gastric gavage tube. Blood samples were collected via the carotid artery at 0, 5, 15, 30, 60, 90, 120, 180, 240, 360, 480 or 600 min after the drug administration. Other procedures were similar to those for the intravenous study.

### 2.4. Effect of Verapamil on Metformin Uptake in HEK-293 Cells Overexpressing OCT1 or OCT2

To investigate whether OCT1 and OCT2, as the main transporters affecting metformin pharmacokinetics, were changed by verapamil, the effect of verapamil on OCT1- or OCT2-mediated metformin uptake was investigated in HEK-293 cells overexpressing OCT1 or OCT2 following previously published procedures [33,34]. Briefly, a density of 4.0 × 10^5^ cells/well of HEK-293 cells overexpressing either OCT1 or OCT2 were seeded into 24-well plates coated with poly-D-lysine (Corning Incorporated, Corning, NY, USA) and incubated with Dulbecco’s Modified Eagle Medium supplemented with 10% fetal bovine serum for 24 h. After washing twice with pre-warmed Hank’s Balanced Salt Solution with Ca^2+^ and Mg^2+^ (Hank’s buffer) and pre-incubating the cells with Hank’s buffer for 10 min, Hank’s buffer was replaced to Hank’s buffer containing 10 μM metformin with verapamil added as an inhibitor (0–100 μM). The concentrations of metformin and verapamil were chosen in the ranges of concentrations used in HEK-293 cells overexpressing OCT1 or OCT2 in the previous reports [33,34] and a protocol of in vitro screening method for human SLC uptake transporter inhibition (OCT1 and OCT2) by Cyprotex. Metformin uptake was initiated at this time and the cells were incubated at 37 °C for 10 min. At 10 min after starting metformin uptake, Hank’s buffer was removed and the cells were immediately washing twice with ice-cold Hank’s buffer to stop metformin uptake. The cells were lysed with distilled water and harvested by scraping them off in 200 μL distilled water followed by ultra-sonification at 4 °C for 10 s. After centrifuging the cells at 15,000× *g* for 10 min at 4 °C, the supernatant was determined by LC/MS/MS analysis of metformin [33,34]. The half-maximal inhibitory constant (IC_50_) values of verapamil for the inhibition of OCT1 or OCT2-mediated metformin uptake are expressed as % of metformin uptake without verapamil (exposed to vehicle instead of verapamil) in HEK-293 cells overexpressing OCT1 or OCT2, arbitrarily set at 100% (*n* = 2 for each dose). From percentages of metformin uptake versus inhibitor concentrations, a sigmoid shaped curve was fitted to the data and IC_50_ was calculated by fitting Hill equation to the data using GraphPad Prism 5 (GraphPad Software Inc., San Diego, CA, USA).

### 2.5. Effect of Verapamil on Metformin Concentration in the Liver and Kidneys

To investigate whether verapamil changes the metformin concentration in the liver and kidneys, a tissue distribution study of metformin with and without verapamil was conducted following a previously reported method [17,33,34]. At 0.5, 1, 3 and 6 h after intravenous or oral administration of metformin with and without verapamil at the same doses as in the pharmacokinetic study, as much blood as possible was collected and each rat was then sacrificed by lethal blood loss. The liver and kidneys were excised, weighed and homogenized in a 4-fold volume of 0.9% NaCl-injectable solution (*n* = 4 for each organ). After centrifuging each homogenate at 15,000× *g* for 10 min at 4 °C, the supernatant was collected and stored at −80 °C.

### 2.6. Effect of Metformin on Verapamil Metabolism in Rat Hepatic and Intestinal Microsomes

To investigate the effect of metformin on verapamil metabolism, the measurement of kinetic constants, such as *V*_max_ (maximum velocity) and *K*_m_ (apparent Michaelis–Menten constant; the concentration at which the rate is one half of the *V*_max_) for verapamil metabolism, with and without metformin, in hepatic and intestinal microsomes were conducted. Hepatic and intestinal microsomes were prepared by the previously reported method as followings [17,37,38]: freshly excised livers were cut in pieces, washed extensively with ice-cold solution (KCl 0.15 M) to remove remaining blood and were homogenized with Tris-HCl buffer (pH 7.5) containing 0.15 M KCl + 50 mM Tris, 1 mM EDTA in a Potter-Elvehjem glass homogenizer for 30 s. The homogenate was centrifuged for 10 min at 10,000× *g* and 4 °C and then followed by ultracentrifugation of the remaining supernatant for 1 h at 100,000× *g* and 4 °C. Microsomal pellets were then re-suspended in the same buffer with a hand homogenizer and re-centrifuged for 1 h at 100,000× *g* and 4 °C. The supernatant was discarded, and the microsomal pellets were carefully overlaid with 0.15 mol KCl buffer and stored at −80 °C. Intestinal microsomes were prepared using freshly excised proximal and middle sections of the small intestine. This part of intestine was excised, rinsed with ice-cold 0.01 M potassium phosphate buffer with 1.15% KCl (pH 7.4), filled with solution A (1.5 mM KCl + 96 mM NaCl + 27 mM sodium citrate + 8 mM KH_2_PO_4_ + 5.6 mM Na_2_HPO_4_ + 40 μg/mL PMSF). The intestine filled with solution A was incubated in a 37 °C water bath for 15 min. After discarding solution A, the intestine was filled with ice-cold solution B (phosphate-buffered saline + 1.5 mM EDTA + 0.5 mM dithiothreitol + 40 μg/mL PMSF), wound around a middle finger and tapped against the finger three times. The upper villus cells were released into solution B during this process, and the collected cells were pooled. The pooled solution was centrifuged at 10× *g* and 4 °C for 5 min. After discarding the supernatant, approximately 15 mL of ice-cold solution C (5 mM histidine + 0.25 M sucrose + 0.5 mM EDTA + 40 μg/mL PMSF) was added into each centrifuge tube, which was inverted twice. Following the discard of the supernatant, the cells were resuspended in fresh ice-cold solution C, homogenized with a Pyrex glass Potter-Elvehjem homogenize and centrifuged at 10,000× *g* and 4 °C for 20 min. The supernatant was then centrifuged at 100,000× *g* and 4 °C for 65 min. The pellet of intestinal microsome was resuspended in 0.2 mM EDTA/20% glycerol/80% 0.1 M phosphate buffer (pH 7.4), homogenized and stored at −80 °C. Protein contents in hepatic and intestinal microsomes were measured by the reported method [39].

In hepatic microsomes (equivalent to 1 mg protein), 2.5 µL of 0.9% NaCl-injectable solution containing 2.5, 5, 10, 20 or 50 µM verapamil (the substrate) as final concentrations, 2.5 µL of 0.9% NaCl-injectable solution containing 10 µM metformin (the inhibitor) as a final concentration and 50 µL of 1 mM of NADPH dissolved in 0.1 M phosphate buffer of pH 7.4 were added. The total volume, 0.5 mL, was adjusted by adding 0.1 M phosphate buffer (pH 7.4), and then the components were incubated at 37 °C using a thermomixer at 500 opm. In intestinal microsomes, verapamil (as the substrate) concentrations of 5, 10, 20, 50 or 200 µM were used and the other conditions were the same as in the hepatic microsome study. After incubation for 15 or 30 min of the hepatic and intestinal microsomes, respectively, 1 mL of diethylether containing 1 µg/mL of propranolol, as an IS, was added to terminate the reaction. The verapamil concentration in each sample was determined by HPLC analysis [29].

The *K*_m_ and *V*_max_ for the verapamil metabolism were calculated using a non-linear regression method [40]. The unweighted kinetic data from the hepatic and intestinal microsomes were fitted using a single-site Michaelis−Menten equation; *V* = *V*_max_ × [S]/(*K*_m_ + [S]), in which [S] was the substrate concentration. The intrinsic clearance (CL_int_) was calculated by dividing the *V*_max_ by the *K*_m_.

### 2.7. Rat Plasma Protein Binding of Metformin and Verapamil Using Equilibrium Dialysis

Protein binding values of metformin and verapamil with and without each other were measured in the fresh plasma of control rats using equilibrium dialysis (*n* = 4 for each; [17]). A 1 mL aliquot of the plasma was dialyzed against 1 mL of isotonic Sørensen phosphate buffer (pH 7.4) containing 3% dextran (*w/v*) in a dialysis cell (Spectrum Medical Industries, Laguna Hills, CA, USA) using a Spectra/Por 4 membrane (mol. wt. cutoff 12–14 KDa; Spectrum Medical Industries, USA). After 24 h incubation, two 50 µL aliquots were collected from each compartment, and the samples were stored at −80 °C.

### 2.8. Analytical Methods for Metformin, Verapamil and Norverapamil

In the HPL-UV system, the metformin concentration in the sample was determined using the analytical method developed by Choi et al. [17]. The quantitation limits of metformin in rat plasma, urine and GI samples were 0.05, 0.1 and 0.1 µg/mL, respectively. The inter- and intra-day coefficients of variation were below 8.94%. Additionally, the metformin concentration in the sample from the metformin uptake study was determined by LC-MS/MS analysis [33,34]. The quantitation limits of metformin in rat plasma, urine and GI samples were 0.01, 0.02 and 0.02 µg/mL, respectively. The inter- and intra-day coefficients of variation were below 9.53%. Verapamil and norverapamil concentrations in the sample were also determined by the HPLC-UV analytical method of Hong et al. [29]. The quantitation limits of verapamil in rat plasma, urine and GI samples were 0.01, 0.05 and 0.05 µg/mL, respectively. The corresponding values of norverapamil in all biological samples was 0.02 µg/mL; the inter- and intra-day coefficients of variation were below 12.9%.

### 2.9. Pharmacokinetic Analysis

Standard methods [41] were used to calculate the following pharmacokinetic parameters using non-compartmental analysis (WinNonlin; Pharsight Corporation, Mountain View, CA, USA): the total area under the plasma concentration–time curve from time zero to infinity (AUC), terminal half-life, time-averaged total body, renal, and non-renal clearances (CL, CL_R_ and CL_NR_, respectively), and apparent volume of distribution at a steady state (V_ss_). The peak plasma concentration (*C*_max_) and time to reach *C*_max_ (*T*_max_) were directly read from the experimental data.

### 2.10. Statistical Analysis

A *p*-value < 0.05 was deemed to be statistically significant using a Student’s *t*-test between the two means for the unpaired data. All results are expressed as mean ± standard deviation (S.D.) except the medians (ranges) for *T*_max_.

## 3. Results

### 3.1. Effect of Verapamil on Metformin Pharmacokinetics

**Figure 1:**
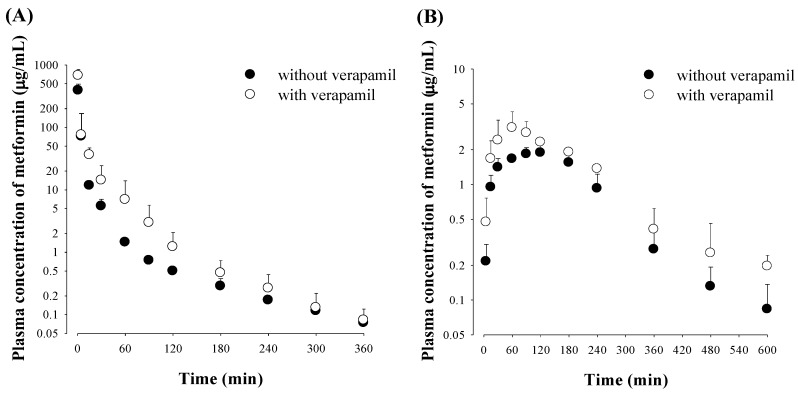
Mean (± S.D.) arterial plasma concentration–time profiles of metformin after intravenous (**A**; *n* = 6 for each) and oral (**B**; *n* = 5 for each) administration of metformin with (○) or without (●) verapamil to rats. Doses of metformin and verapamil were 30 and 20 mg/kg, respectively.

The mean arterial plasma concentration–time profiles and relevant pharmacokinetic parameters of metformin after its intravenous administration with and without verapamil are shown in Figure 1 and Table 1, respectively. After intravenous administration of metformin with verapamil, the AUC was significantly greater (by 66.7%); CL and CL_R_ were significantly slower (by 50.8 and 70.7%, respectively); and Ae_0–24 h_ was significantly smaller (by 43.0%) than those without verapamil.

After oral administration of metformin with verapamil, the AUC was significantly greater (by 73.5%), *C*_max_ was significantly higher (by 60.5%), CL_R_ was significantly slower (by 68.4%) and Ae_0–24 h_ was significantly smaller (by 46.0%) than those without verapamil.

### 3.2. Effect of Verapamil on OCT1- or OCT2-Mediated Metformin Uptake in HEK-293 Cells Overexpressing OCT1 or OCT2

**Figure 2:**
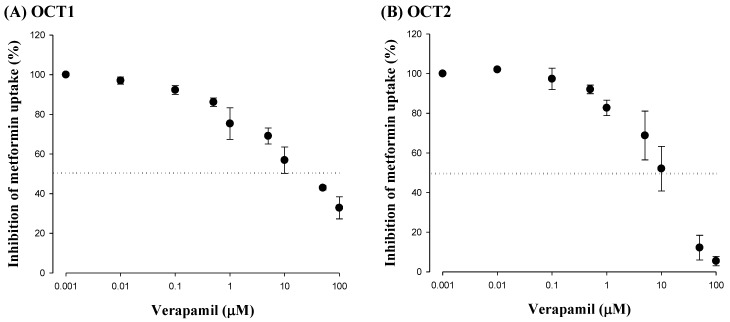
Mean (± S.D.) inhibitory effect of verapamil on metformin uptake in HEK293 cells overexpressing OCT1 (**A**, *n* = 2) or OCT2 (**B**, *n* = 2).

To examine the verapamil effect on OCT1 and OCT2 activities, the changes of metformin uptake with and without verapamil were compared in HEK-293 cells overexpressing OCT1 or OCT2. As shown in Figure 2, verapamil considerably inhibited metformin uptake in HEK-293 cells overexpressing either OCT1 or OCT2. The IC_50_ values of verapamil for inhibiting OCT1- and OCT2-mediated metformin uptake were 51.9 ± 1.09 and 19.3 ± 0.220 μM, respectively. 

### 3.3. Effect of Verapamil on Metformin Concentrations in the Liver and Kidneys

**Figure 3:**
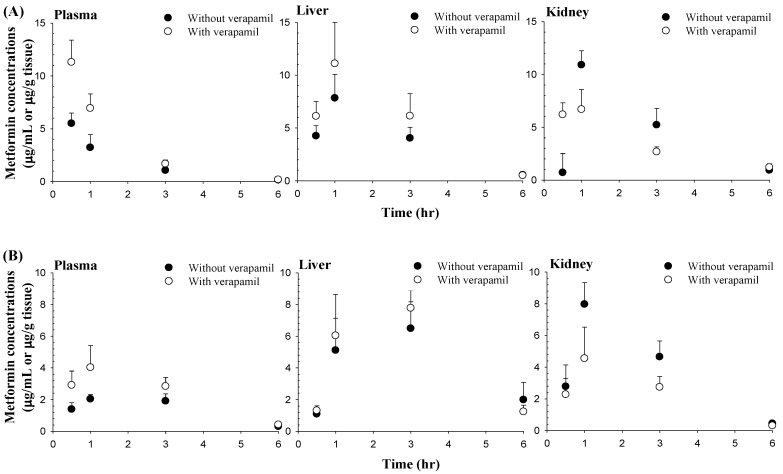
Mean (± S.D.) concentrations of metformin in the plasma, liver and kidneys after its intravenous (**A**, *n* = 4 for each) or oral (**B**, *n* = 4 for each) administration with (○) or without (●) verapamil. The dose of metformin and verapamil were 30 and 20 mg/kg, respectively.

After intravenous and oral administration of metformin with and without verapamil, metformin concentrations in the liver and kidneys are shown in Figure 3 and Table 2, respectively. After intravenous and oral administration of both drugs together, verapamil did not cause any change of the metformin concentration in the liver, but the metformin concentration in the kidneys was significantly decreased (by 38.6 and 48.7% at 1 and 3 h in the intravenous study and 42.9, 41.1 and 30.2% at 1, 3 and 6 h in the oral study, respectively) compared to metformin alone. Metformin concentrations in the plasma after intravenous and oral co-administration of metformin and verapamil were significantly lower than those after administration of metformin alone, showing similar patterns to the pharmacokinetic study.

### 3.4. Effect of Metformin on Verapamil Pharmacokinetics

**Figure 4:**
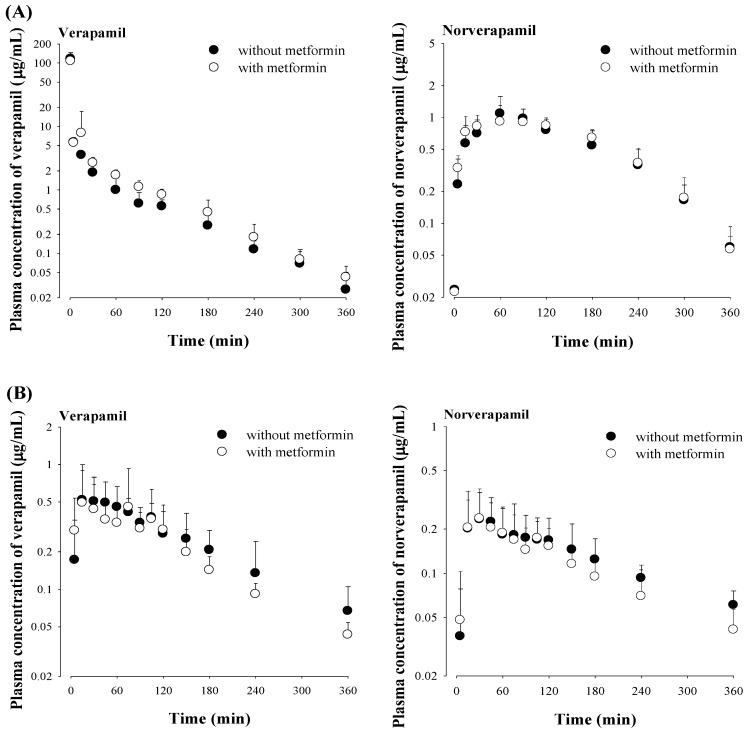
Mean (± S.D.) arterial plasma concentration–time profiles of verapamil and norverapamil after intravenous (**A**, *n* = 6 for each) or oral (**B**, *n* = 5 for each) administration of verapamil with (○) or without (●) metformin to rats. The doses of metformin and verapamil were 30 and 20 mg/kg, respectively.

The mean arterial plasma concentration–time profiles and relevant pharmacokinetic parameters of verapamil and norverapamil after intravenous administration of verapamil with and without metformin are shown in Figure 4 and Table 3, respectively. After intravenous and oral administration of verapamil with metformin, all pharmacokinetic parameters of verapamil and norverapamil including the AUC_norverapamil_/AUC_verapamil_ ratio were comparable to those without metformin. The absorption of verapamil from the gastrointestinal tract and the formation of norverapamil were rapid based on the *C*_max_ of verapamil and norverapamil, respectively.

### 3.5. Effect of Metformin on Verapamil Metabolism in Rat Hepatic and Intestinal Microsomes

**Figure 5:**
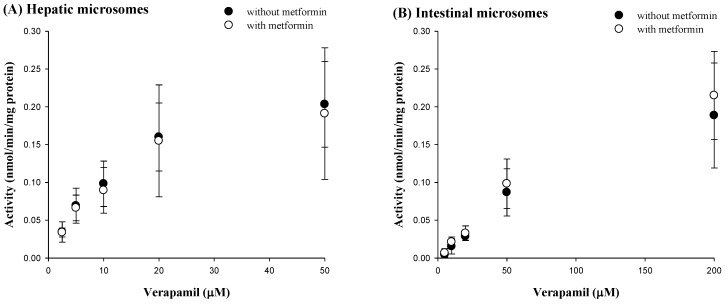
Nonlinear regression for mean values (± S.D.) of verapamil metabolism activity with (○) or without (●) metformin in rat hepatic (**A**, *n* = 5 for each) or intestinal (**B**, *n* = 4 for each) microsomes.

The V_max_, K_m_ and CL_int_ for the metabolism of verapamil with and without metformin in rat hepatic and intestinal microsomes are shown in Figure 5 and Table 4, respectively. There was no change of V_max_, K_m_ or CL_int_ for verapamil metabolism in the presence of metformin, indicating that metformin did not affect verapamil metabolism in rat hepatic and intestinal microsomes under these conditions.

### 3.6. Rat Plasma Protein Binding of Verapamil and Metformin Using Equilibrium Dialysis

The concentration of 5 g/mL of each drug was chosen based on previous reports [17,42]. Protein binding values of metformin with and without verapamil were 13.5% ± 5.39% and 11.6% ± 6.10%, respectively. The corresponding values for verapamil with and without metformin were 93.0% ± 38.9% and 85.1% ± 26.3%, respectively. Metformin and verapamil did not affect the rat plasma protein binding to each other.

## 4. Discussion

The ratio of AUC of a drug with an inhibitor (AUC_i_)/ AUC of a drug without an inhibitor (AUC_0_) of over 1.25 is classified as a relevant drug interaction by an inhibitor in U.S. FDA criteria [43]. In our study, the ratios of AUC_i_/AUC of metformin with and without verapamil were 1.67 and 1.74 (i.e., relative bioavailability) after the intravenous and oral administration of both drugs (Table 1), indicating that verapamil might act as an inhibitor to cause a pharmacokinetic interaction with metformin.

In the intravenous study, the contribution of CL_R_ to CL, 66.5%, was a large portion of the metformin elimination pathway (Table 1), indicating that renal excretion is the main route of metformin elimination. The estimated CL_R_s of metformin considering the free fractions of metformin in the plasma (CL_R_,_fu_s) were 4.08 and 14.2 mL/min/kg with and without verapamil, respectively. The CL_R_,_fu_ of metformin without verapamil was faster than the reported glomerular filtration rate (GRF, represented by creatinine clearance), 5.24 mL/min/kg, in rats [44], indicating that active secretion of metformin as its renal excretion mechanism is changed by verapamil to glomerular filtration. Verapamil slowed the CL_R_,_fu_ of metformin, 4.08 mL/min/kg, to the creatinine clearance level in rats, indicating that verapamil might cause metformin reabsorption in the renal tubules. The inhibited renal excretion pathway caused a dramatic increase in the systemic exposure (e.g., AUC) of metformin in this study similar as other references [18,45]. 

To investigate the inhibitory mechanism of verapamil on renal excretion of metformin, the IC_50_ of verapamil against metformin uptake in HEK-293 cells overexpressing OCT2 was conducted based on the known facts that metformin is an OCT2 substrate and verapamil inhibits OCT2 [15,16]. The inhibitory effect of verapamil on OCT2-mediated metformin uptake in HEK-293 cells overexpressing OCT2 (Figure 2) supported the reduced CL_R_ of metformin when co-administered with verapamil (Table 1). Verapamil significantly reduced the metformin concentration in the kidneys after intravenous and oral administration of metformin with verapamil (Table 2 and Figure 3), probably due to the inhibition of OCT2-mediated metformin uptake into the proximal renal tubules, as shown in the IC_50_ of verapamil in HEK-293 cells over-expressing OCT2 (Figure 2).

Considering that the metformin concentration in the liver is important to preserve the glucose-lowering effect of metformin [11,46,47], the metformin concentration in the liver was also measured after intravenous and oral administration of both drugs (Table 2 and Figure 3). However, the metformin concentration in the liver was not changed by verapamil, which might be due to verapamil not sufficiently inhibiting OCT1-mediated metformin uptake in the sinusoidal membrane of hepatocytes. Since OCT1 in the basolateral membrane uptakes metformin from the sinusoidal blood into hepatocytes, comparable metformin concentrations in the liver with and without verapamil could be supported by the relatively high IC_50_ of verapamil for inhibiting metformin uptake by HEK-293 cells over-expressing OCT1 (Figure 2). In other words, verapamil might have a stronger potential to inhibit OCT2 activity than OCT1 activity. Although verapamil inhibited OCT1 and OCT2-mediated metformin uptake in vitro (Figure 2), the inhibitory effect of verapamil on OCT1-mediated metformin uptake in hepatocytes might be almost negligible in in vivo studies (Table 2 and Figure 3). Therefore, verapamil is an OCT2 inhibitor in the renal proximal tubules, resulting in reduced renal excretion and increased systemic exposure of metformin.

On the other hand, the contribution of gastrointestinal (including biliary) excretion of unchanged metformin to its CL_NR_ was almost negligible; the GI_24 h_ was less than 0.471% of the intravenous dose (Table 1). Similarly, it has been reported that metformin is mainly eliminated via renal excretion, but the biliary excretion of metformin as a parent form into feces was negligible [11,17]. In the aspect of metformin and verapamil interactions, the unchanged CL_NR_ and GI_24 h_ of metformin by verapamil indicted that verapamil did not influence the non-renal elimination pathway (e.g., biliary excretion and metabolism) of metformin in rats. 

After oral administration of metformin with and without verapamil, oral absorption of metformin was rapid and extensive regardless of co-administration of verapamil. For comparison, we estimated the mean ‘true’ unabsorbed fractions (‘*F*_unabs_’) after oral metformin administration to rats with and without verapamil based on the following reported equation [48]:

0.0357 = ‘*F*_unabs_’ + (0.00471 × 0.297)   without verapamil 0.0153 = ‘*F*_unabs_’ + (0.00831 × 0.309)   with verapamil (1)

where 0.0357 (0.0153), 0.00471 (0.00831) and 0.297 (0.309) are the oral GI_24 h_, intravenous GI_24 h_ and *F*, respectively, of metformin without verapamil (with verapamil). The ‘*F*_unabs_’ values thus estimated were 1.27% and 3.43% with and without verapamil, respectively, indicating that verapamil probably does not affect metformin absorption in the intestine (Table 1). Thus, the reduced AUC of metformin with verapamil could be due to the inhibition of renal excretion of metformin by verapamil, for the same reason as in the intravenous study.

In contrast, metformin did not change any pharmacokinetic profile of verapamil after intravenous and oral administration of metformin and verapamil together compared to verapamil alone. Metformin also did not affect the formation of norverapamil as an active metabolite of verapamil (Table 3). As hepatic metabolism via CYP3A is the main route of elimination of verapamil [28,49], and a suppressive effect of metformin on PXR-regulating CYP3A4 has been reported [21], the change of verapamil metabolism by metformin was evaluated with greater focus in this study. In parallel to the unchanged CL_NR_ of verapamil by metformin in the intravenous co-administration of metformin and verapamil (Table 3), metformin showed a negligible interaction with verapamil metabolism in in vitro hepatic and microsomal studies (Table 4). Considering that verapamil is a drug with a high (or intermediate) hepatic extraction ratio in rats [44], its hepatic clearance (metabolism) depends on the hepatic CL_int_ and free fraction (unbound to plasma proteins) of verapamil, and hepatic blood flow rate [50]. In our study, the unchanged CL_NR_s of verapamil with and without metformin (Table 3) were supported by the comparable hepatic CL_int_s and free unfound fractions of verapamil, and constant hepatic blood flow rate with and without metformin in rats. Metformin might not affect the hepatic blood flow rate based on studies in humans [51]. In other words, these findings indicated that metformin might inhibit the hepatic metabolism of verapamil including the formation of norverapamil (Table 3). Additionally, the unchanged *K*_m_s and *V*_max_s of verapamil with metformin compared to those without metformin in in vitro hepatic microsomal studies indicated that metformin did not affect the affinity between metabolic enzyme and verapamil and maximum rate of metabolism of verapamil. 

Although the contribution of renal excretion of verapamil is minor in regards to its elimination, the renal excretion of verapamil with and without metformin was estimated as follows: the CL_R,fu_s of verapamil with and without metformin adjusted by the free fraction of verapamil in the plasma were 0.00756 and 0.219 mL/min/kg, respectively. Both CL_R,fu_s of verapamil were significantly slower than the reported GFR, indicating that glomerular filtration was a renal excretion mechanism of verapamil regardless of the presence of metformin and metformin did not inhibit its renal excretion in rats (Table 3).

After oral administration of both drugs, no effect of metformin on the pharmacokinetic profiles of verapamil and norverapamil was observed. The comparable AUCs of verapamil with and without metformin (Table 3) were likely due to the unchanged absorption of verapamil; the estimated ‘*F*_unabs_’ values of verapamil were 0.00179 and 0.00256% for with and without metformin, respectively, from the equations [48]:

0.00291 = ‘*F*_unabs_’ + (0.00148 × 0.234)   without metformin 0.00200 = ‘*F*_unabs_’ + (0.00117 × 0.182)   with metformin (2)

where 0.00291 (0.00200), 0.00148 (0.00117) and 0.234 (0.182) are the oral GI_24 h_, intravenous GI_24 h_ and *F* of verapamil, respectively, without metformin (with metformin). Thus, metformin might not affect verapamil absorption in the intestine (Table 3). Orally administered metformin might also not inhibit verapamil metabolism in the liver and intestine, as supported by the unchanged verapamil metabolism by metformin in the in vitro hepatic and intestinal microsome studies (Table 4). 

Although Cho et al. [32] reported the inhibitory effect of verapamil on the glucose tolerance activity of metformin without any change of metformin’s AUC in healthy adults, verapamil has recently emerged as a new indication for the treatment of hypertension in DM patients [22,23]. Extrapolating the rat dose to human equivalent dose based on the equation by FDA [52], there is a slight difference: the estimated human equivalent doses of metformin and verapamil in our study are 340 mg/70 kg and 227 mg/70 kg, respectively, and the corresponding doses in Cho et al. [32] were 1000 mg and 180 mg per patient (average body weight of patients was 70.5 kg). This inconsistency of doses can be one reason for the result in the different PK interaction pattern in metformin and verapamil combination. In addition, the relatively lower doses were used to emphasize the potential for the occurrence of metformin and verapamil interaction, which can provide a clue to cause OCT mediated drug interaction as underlying mechanism for the further investigations using various dosage regimens in preclinical as well as clinical studies.

## 5. Conclusions

After intravenous and oral administration of both drugs, the significantly greater AUC of metformin could be due to the inhibition of OCT2-mediated renal excretion of metformin by verapamil, leading to increased systemic exposure of metformin. Interestingly, there was no interaction effect on the metformin concentration in the liver in spite of the inhibitory effect of verapamil on OCT1-mediated metformin uptake in vitro. In contrast, metformin did not influence the pharmacokinetic profile of verapamil. These results can provide essential knowledge about the drug interaction potential between metformin and verapamil for their clinical applications.

## Figures and Tables

**Table 1:** Mean (± S.D.) pharmacokinetic parameters of metformin after its intravenous and oral administration with and without verapamil to rats. Doses of metformin and verapamil were 30 and 20 mg/kg, respectively.

Parameter	Intravenous (*n* = 6 for Each)		Parameter	Oral (*n* = 5 for Each)	
	Without Verapamil	With Verapamil		Without Verapamil	With Verapamil
Body weight (g)	203 ± 8.80	209 ± 6.65	Body weight (g)	228 ± 19.2	217 ± 13.0
AUC (μg min/mL)	1590 ± 613	2650 ± 449 ^a^	AUC (μg min/mL)	472 ± 40.2	819 ± 82.3 ^b^
Terminal half-life (min)	92.4 ± 26.4	80.5 ± 34.6	Terminal half-life (min)	137 ± 44.4	101 ± 48.0
MRT (min)	21.4 ± 5.28	18.4 ± 7.69	*C*_max_ (μg/mL)	2.00 ± 0.328	3.21 ± 1.04 ^c^
V_SS_ (mL/kg)	349 ± 128	286 ± 92.4	*T*_max_ (min)	60 (60–120)	60 (60–120)
CL (mL/min/kg)	24.2 ± 3.02	11.9 ± 2.00 ^b^	CL_R_ (mL/min/kg)	45.6 ± 5.66	14.4 ± 5.45 ^b^
CL_NR_ (mL/min/kg)	8.12 ± 3.03	7.13 ± 0.932	Ae_0–24 h_ (% of dose)	71.3 ± 5.86	38.5 ±9.96 ^b^
CL_R_ (mL/min/kg)	16.1 ± 3.18	4.72 ± 2.52 ^b^	GI_24 h_ (% of dose)	3.57 ± 2.84	1.53 ± 1.57
Ae_0–24 h_ (% of dose)	66.5 ± 11.3	37.9 ± 14.9 ^a^	*F* (%)	29.7	30.9
GI_24 h_ (% of dose)	0.471 ± 0.782	0.831 ± 0.787			

AUC, total area under the plasma concentration−time curve from time zero to infinity; MRT, mean residence time; V_ss_, apparent volume of distribution at steady state; CL, time-averaged total body clearance; CL_NR_, time-averaged non-renal clearance; CL_R_, time-averaged renal clearance; Ae_0–24 h_, percentage of the dose excreted in the urine up to 24 h; GI_24 h_, percentage of the dose recovered from the gastrointestinal tract (including its contents and feces) at 24 h; *C*_max_, peak plasma concentration of docetaxel; *T*_max_, time to reach *C*_max_; *F*, extent of absolute oral bioavailability. ^a^ Significantly different (*p* < 0.01) from with verapamil. ^b^ Significantly different (*p* < 0.001) from with verapamil. ^c^ Significantly different (*p* < 0.05) from with verapamil.

**Table 2:** Mean (± S.D.) concentrations (μg/mL or μg/g tissue) of metformin in the plasma, liver and kidneys after intravenous or oral administration of metformin with and without verapamil.

	Intravenous (*n* = 4 for Each)		Oral (*n* = 4 for Each)	
Parameter	Without Verapamil	With Verapamil	Without Verapamil	With Verapamil
**Plasma**				
0.5	5.50 ± 0.988	11.3 ± 2.09 ^a^	1.39 ± 0.415	2.91 ± 0.894 ^b^
1	3.22 ± 1.22	6.94 ± 1.34 ^a^	2.04 ± 0.281	4.03 ± 1.38 ^b^
3	1.06 ± 0.214	1.68 ± 0.377 ^b^	1.91 ± 0.455	2.84 ± 0.564 ^b^
6	0.158 ± 0.0530	0.184 ± 0.0789	0.306 ± 0.0724	0.421 ± 0.0570 ^b^
**Liver**				
0.5	4.25 ± 0.978	6.12 ± 1.39	1.09 ± 0.207	1.31 ± 0.300
1	7.84 ± 2.25	11.1 ± 3.84	5.11 ± 2.03	6.04 ± 2.60
3	4.04 ± 1.04	6.14 ± 2.10	6.49 ± 1.68	7.78 ± 1.11
6	0.553 ± 0.108	0.510 ± 0.222	1.99 ± 1.10	1.24 ± 0.407
**Kidney**				
0.5	0.706 ± 1.80	6.20 ± 1.12	2.78 ± 1.36	2.28 ± 1.01
1	10.9 ± 1.34	6.69 ± 1.87 ^b^	7.97 ± 1.36	4.55 ± 1.98 ^b^
3	5.22 ± 1.57	2.68 ± 0.471 ^b^	4.65 ± 1.01	2.74 ± 0.675 ^b^
6	0.935 ± 0.284	1.20 ± 0.342	0.443 ± 0.0640	0.309 ± 0.0767 ^b^

^a^ Significantly different (*p* < 0.01) from metformin. ^b^ Significantly different (*p* < 0.05) from metformin.

**Table 3:** Mean (± S.D.) pharmacokinetic parameters of verapamil and norverapamil after intravenous or oral administration of verapamil without and with metformin to rats. The doses of metformin and verapamil were 30 and 20 mg/kg, respectively.

Parameter	Intravenous (*n* = 6 for Each)		Parameter	Oral (*n* = 5 for Each)	
	Without Metformin	With Metformin		Without Metformin	With Metformin
**Verapamil**			**Verapamil**		
Body weight (g)	205 ± 8.37	209 ± 6.65	Body weight (g)	214 ± 9.62	217 ± 13.0
AUC (μg min/mL)	441 ± 69.5	527 ± 134	AUC (μg min/mL)	103 ± 21.6	96.0 ± 30.6
Terminal half-life (min)	71.1 ± 19.0	79.1 ± 24.7	Terminal half-life (min)	128 ± 59.1	121 ± 57.5
MRT (min)	40.9 ± 7.75	42.4 ± 20.4	*C*_max_ (μg/mL)	0.678 ± 0.386	0.714 ± 0.452
V_SS_ (mL/kg)	1600 ± 861	1860 ± 535	*T*_max_ (min)	45 (15–105)	30 (15–75)
CL (mL/min/kg)	46.3 ± 7.14	40.0 ± 9.45	CL_R_ (mL/min/kg)	0.671 ± 0.748	0.489 ± 0.256
CL_NR_ (mL/min/kg)	46.1 ± 7.08	39.8 ± 9.40	Ae_0–24 h_ (% of dose)	0.308 ± 0.215	0.231 ± 0.102
CL_R_ (mL/min/kg)	0.147 ± 0.121	0.108 ± 0.0726	GI_24 h_ (% of dose)	0.291 ± 0.339	0.200 ± 0.135
Ae_0–24 h_ (% of dose)	0.306 ± 0.219	0.258 ± 0.125	*F* (%)	23.4	18.2
GI_24 h_ (% of dose)	0.148 ± 0.201	0.117 ± 0.0759			
**Norverapamil**			**Norverapamil**		
AUC (μg min/mL)	188 ± 34.7	199 ± 38.7	AUC (μg min/mL)	68.3 ± 38.6	53.9 ± 24.8
Terminal half-life (min)	50.8 ± 10.2	63.3 ± 15.2	Terminal half-life (min)	168 ± 73.2	117 ± 44.5
*C*_max_ (μg/mL)	1.24 ± 0.386	1.09 ± 0.308	*C*_max_ (μg/mL)	0.267 ± 0.141	0.273 ± 0.123
*T*_max_ (min)	60 (30−120)	60 (30−90)	*T*_max_ (min)	45 (15–120)	45 (15–105)
CL_R_ (mL/min/kg)	0.109 ± 0.102	0.0626 ± 0.0535	CL_R_ (mL/min/kg)	0.336 ± 0.107	0.562 ± 0.445
Ae_0–24 h_ (% of dose)	0.0872 ± 0.0691	0.0585 ± 0.0453	Ae_0–24 h_ (% of dose)	0.104 ± 0.0567	0.113 ± 0.0522
GI_24 h_ (% of dose)	0.146 ± 0.0841	0.126 ± 0.0927	GI_24 h_ (% of dose)	0.0870 ± 0.0665	0.0782 ± 0.0514
AUC_norverapamil_/AUC_verapamil_	43.0 ± 7.68	38.5 ± 11.6	AUC_norverapamil_/AUC_verapamil_	87.3 ± 26.7	89.7 ± 27.0

AUC, total area under the plasma concentration−time curve from time zero to infinity; MRT, mean residence time; V_ss_, apparent volume of distribution at steady state; CL, time-averaged total body clearance; CL_NR_, time-averaged non-renal clearance; CL_R_, time-averaged renal clearance; Ae_0–24 h_, percentage of the dose excreted in the urine up to 24 h; GI_24 h_, percentage of the dose recovered from the gastrointestinal tract (including its contents and feces) at 24 h; *C*_max_, peak plasma concentration of docetaxel; *T*_max_, time to reach *C*_max_; *F*, extent of absolute oral bioavailability.

**Table 4:** Mean (± S.D.) *K*_m_, *V*_max_ and CL_int_ for the metabolism of verapamil with and without metformin in hepatic and intestinal microsomes.

Parameter	Without Metformin	With Metformin
**Hepatic**	***n* = 5 for Each**	
*K*_m_ (μM)	11.0 ± 3.11	13.8 ± 1.26
*V*_max_ (nmol/min/mg protein)	0.225 ± 0.0951	0.289 ± 0.151
CL_int_ (μL/min/mg protein)	0.0199 ± 0.00419	0.0205 ± 0.00911
**Intestinal**	***n* = 4 for Each**	
*K*_m_ (μM)	148 ± 53.5	171 ± 11.4
*V*_max_ (nmol/min/mg protein)	0.332 ± 0.125	0.412 ± 0.0908
CL_int_ (μL/min/mg protein)	0.00181 ± 0.00150	0.00240 ± 0.000409

*K*_m_, the concentration at which the rate is one-half of the *V*_max_; *V*_max_, maximum velocity; CL_int_, intrinsic clearance.
